# Bud phenology and growth are subject to divergent selection across a latitudinal gradient in *Populus angustifolia* and impact adaptation across the distributional range and associated arthropods

**DOI:** 10.1002/ece3.2222

**Published:** 2016-06-10

**Authors:** Luke M. Evans, Sobadini Kaluthota, David W. Pearce, Gerard J. Allan, Kevin Floate, Stewart B. Rood, Thomas G. Whitham

**Affiliations:** ^1^ Department of Biological Sciences & Merriam‐Powell Center for Environmental ResearchNorthern Arizona UniversityPO Box 5640FlagstaffArizona86011; ^2^ Biological ScienceUniversity of LethbridgeLethbridgeAlbertaT1K 3M4Canada; ^3^ Lethbridge Research and Development CentreAgriculture and Agri‐Food CanadaLethbridgeAlbertaT1J 4B1Canada

**Keywords:** Climate change, cline, cottonwood, ecological community, *F*_ST_, local adaptation, *Q*_ST_

## Abstract

Temperate forest tree species that span large geographical areas and climatic gradients often have high levels of genetic variation. Such species are ideal for testing how neutral demographic factors and climate‐driven selection structure genetic variation within species, and how this genetic variation can affect ecological communities. Here, we quantified genetic variation in vegetative phenology and growth traits in narrowleaf cottonwood, *Populus angustifolia*, using three common gardens planted with genotypes originating from source populations spanning the species' range along the Rocky Mountains of North America (*ca*. 1700 km). We present three main findings. First, we found strong evidence of divergent selection (*Q*_ST_ > *F*_ST_) on fall phenology (bud set) with adaptive consequences for frost avoidance. We also found evidence for selection on bud flush duration, tree height, and basal diameter, resulting in population differentiation. Second, we found strong associations with climate variables that were strongly correlated with latitude of origin. More strongly differentiated traits also showed stronger climate correlations, which emphasizes the role that climate has played in divergent selection throughout the range. We found population × garden interaction effects; for some traits, this accounted for more of the variance than either factor alone. Tree height was influenced by the difference in climate of the source and garden locations and declined with increasing transfer distance. Third, growth traits were correlated with dependent arthropod community diversity metrics. *Synthesis*. Overall, we conclude that climate has influenced genetic variation and structure in phenology and growth traits and leads to local adaptation in *P. angustifolia*, which can then impact dependent arthropod species. Importantly, relocation of genotypes far northward or southward often resulted in poor growth, likely due to a phenological mismatch with photoperiod, the proximate cue for fall growth cessation. Genotypes moved too far southward suffer from early growth cessation, whereas those moved too far northward are prone to fall frost and winter dieback. In the face of current and forecasted climate change, habitat restoration, forestry, and tree breeding efforts should utilize these findings to better match latitudinal and climatic source environments with management locations for optimal future outcomes.

## Introduction

Climate has strong effects on forest health, growth, and productivity (Rehfeldt et al. 1999; van Mantgem et al. 2009; Allen et al. 2010; Wang et al. 2010; Grady et al. 2011, 2015; Urban 2015) and is an important driver of natural selection for many species (Sthultz et al. 2009; Hoffmann and Sgrò 2011; Alberto et al. 2013). Many forest trees have a broad distribution, harbor considerable genetic variation, and are often locally adapted across their range (Clark et al. 2007; Savolainen et al. 2007; O'Neill et al. 2008; Hereford 2009; Richardson et al. 2014). For these reasons, they are well suited for studies of climate impacts on adaptation and productivity. They are important sources of wood for building materials, paper, and energy (Bonan 2008; Difazio et al. 2011; Neale and Kremer 2011) and are often foundation species that drive diversity and structure in ecological communities and associated ecosystem processes (Rood et al. 2003; Whitham et al. 2006; Floate et al. 2016). Understanding the factors that influence patterns of genetic variation within forest trees can lead to insights into adaptation (Linnen and Hoekstra 2009; Barrett and Hoekstra 2011), with important applications for conservation, restoration, and forest management (O'Neill et al. 2008; Wang et al. 2010; Grady et al. 2011; Grady in press).

Vegetative phenology, the timing of growth initiation in the spring and the cessation of growth in the late summer and fall, constitutes a key suite of adaptive traits for temperate zone forest trees with fundamental tradeoffs (Howe et al. 2003; Hereford 2009; Savolainen et al. 2013). Trees that initiate growth too early risk damage from late spring frost, but may gain growing season length. Conversely, those that cease growth earlier in the fall trade growing season length for reduced risk of fall frost damage. Because climatic conditions vary across a species' range, such tradeoffs may result in divergent selection leading to fine‐tuning of the timing of phenological events and adaptation of populations to local environments (Howe et al. 2003; Savolainen et al. 2007). Such phenology and productivity differences across a species' range may influence numerous dependent species, leading to changes in arthropod communities (Mopper 2005; van Asch and Visser 2007; Ikeda et al. 2014).

This relationship between climate and its impact on phenology and growth leads to several testable hypotheses. First, if a trait is under divergent selection, quantitative trait population differentiation described as “*Q*_ST_” is expected to be greater than neutral locus differentiation (*F*_ST_), which summarizes the population differentiation due to demography and stochastic processes using molecular marker data (Spitze 1993; Leinonen et al. 2008). Second, if selection is due to an environmental gradient (e.g., climate), then the trait under selection should also show a clinal relationship with that gradient (Barton 1999). Transfer functions (Wang et al. 2010) test this hypothesis by determining whether tree populations grow best in their local climate. Third, there should be a correlation between the strength of population differentiation and the strength of the relationship between a trait and one or more climate variables. Finally, if climatic variables have resulted in divergent selection on traits that affect productivity, genetic differences in these traits should impact plant productivity/community diversity relationships (Mittelbach et al. 2001; Ikeda et al. 2014). If correct, the ecological and evolutionary consequences of climate change may extend well beyond the focal plants to their associated communities.

The genus *Populus* (Salicaceae) comprises 30–40 species of mostly temperate zone forest trees (Eckenwalder 1996). Phenological and growth traits are genetically based within the genus (Wu et al. 1998; Frewen et al. 2000; Howe et al. 2000) and, in several species, there is evidence of adaptive population differentiation and clinal variation (Pauley and Perry 1954; Dunlap and Stettler 1996; Hall et al. 2007; Rood et al. 2007; Grady et al. 2011; Rohde et al. 2011; Evans et al. 2014; McKown et al. 2014); however, many of these studies included only single plantation locations, limiting the ability to understand genotype x environment interactions. Narrowleaf cottonwood, *Populus angustifolia* James (Rood et al. 2010), shows strong neutral genetic structure throughout its range, with extensive latitudinal clines and population differentiation (Evans et al. 2015; Kaluthota et al. 2015). It is a foundation species that drives dependent communities and ecosystem processes (Whitham et al. 2006), but no published studies have examined phenological trait variation across populations of this species and potential impacts on associated arthropods.

Using replicated common gardens across the range of *P. angustifolia*, we test the hypothesis that key phenological and growth traits are subject to divergent selection and adaptive differentiation along a latitudinal and climatic gradient. Specifically, we ask: (1) Are patterns of quantitative trait differentiation as measured by *Q*_ST_ consistent with divergent selection and do genotype × environment (G × E) interactions influence adaptive traits? (2) Do patterns of trait variation correlate with climate variables, and does altered climate result in altered growth? (3) Do more differentiated traits show stronger relationships with climate variables than less differentiated traits? (4) Do phenological and productivity differences among tree populations and genotypes influence associated arthropod diversity? Answers to these questions provide important insight into whether growth and phenology are subject to divergent selection in *P*. *angustifolia*, with implications for associated communities and the management of widespread forest trees in the face of climate change.

## Materials and Methods

### Collections and common gardens

**Figure 1:**
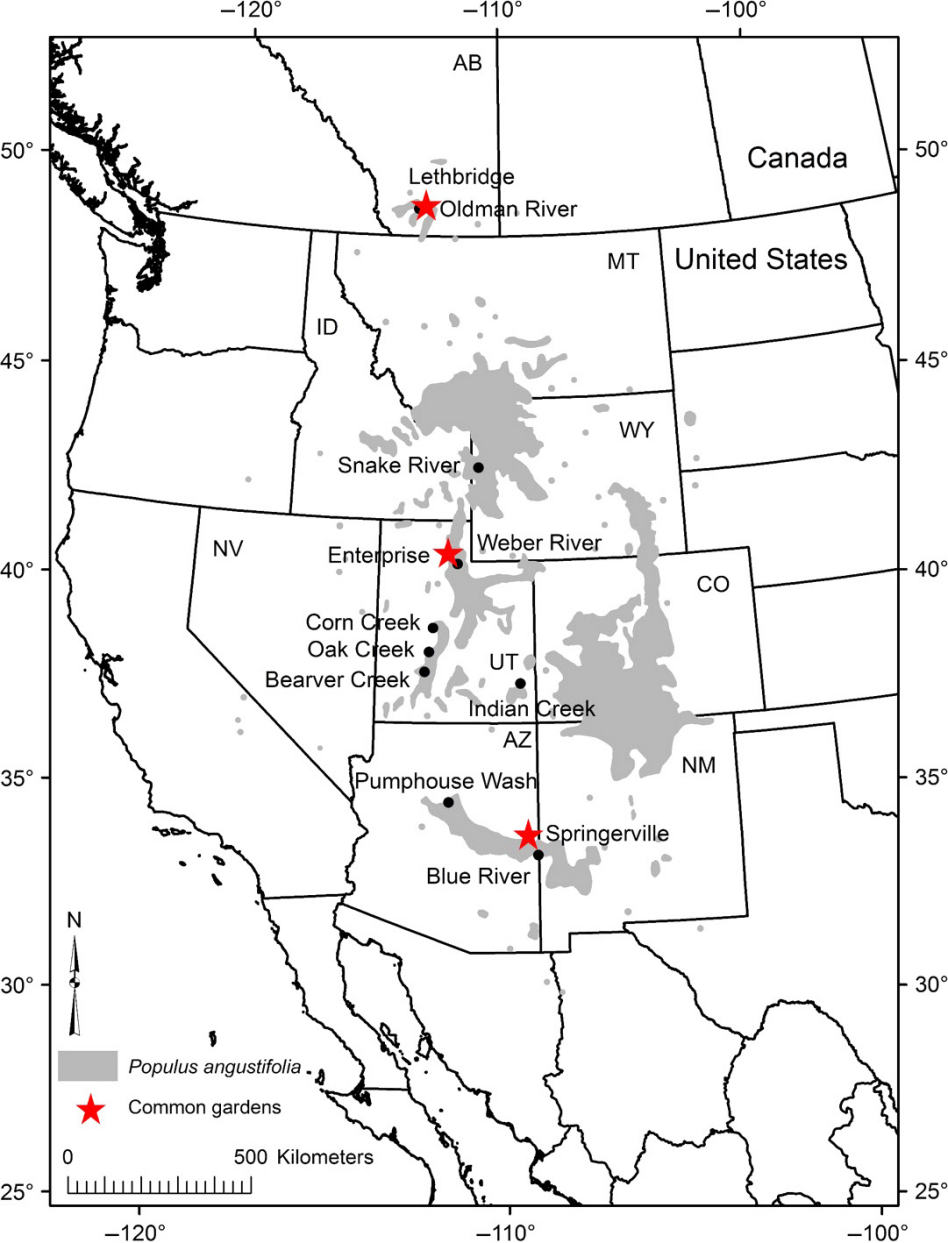
Map of collection locations and planting sites throughout the range of *Populus angustifolia*.

In January and February 2009, we collected vegetative cuttings from 40 to 60 trees along nine rivers (populations) along the north–south distribution (*ca*. 1700 km/15.9° latitude) of *P. angustifolia* (Fig. 1). We pruned cuttings to 1 or 2 live buds each, planted them individually in pots at the Northern Arizona University Research Greenhouse Facility in Flagstaff, AZ, and watered and fertilized as needed. In June and July 2009, we planted three replicated, two‐acre common gardens at the northern (Lethbridge, AB), center (Enterprise, UT), and southern (Springerville, AZ) distribution of the species' range (Fig. 1). The AZ and UT sites were fenced to prevent deer and livestock damage. Trees were spaced 1.5–3.0 m apart and watered as needed. Plants that initially died were replanted in the summer of 2010. Genotyping of each collected tree confirmed that there were no clonal members and this also prompted the rejection of a few collected trees that were determined to be natural interspecific hybrids of *P. angustifolia* and another local cottonwood (Evans et al. 2015).

We planted trees in a completely randomized design. Of the 30–60 genotypes we collected per river, we planted one replicate of every genotype at each garden location. From the three closest collection rivers to the planting sites (“local” rivers; Oldman, Weber, and Blue; Fig. 1), we planted an additional five clonal replicates of 20 genotypes from each local river at all three sites (Kaluthota et al. 2015). In the available garden space, this allowed us to estimate among‐population variation using all nine populations, and also among‐genotype (within‐population) variation using the replicated genotypes spanning the three local rivers. Final sample sizes after initial mortality are summarized in Table S1. However, replanting and mortality altered these sample sizes somewhat for measurements made in different seasons and years.

For each collection location, we used ClimateWNA (Wang et al. 2012) to obtain 1961–1990 climate averages and study year climate for 21 different climate variables. Because many of these variables are correlated across the latitudinal gradient of our sample, we used principal components analysis, decomposing the correlation matrix and including source latitude, longitude, and elevation, in order to produce composite environment variables (Table S2). PC loadings indicate PC1 was primarily driven by variation in temperature and degree‐day variables, whereas PC2 was more strongly influenced by geography (latitude, longitude, and elevation) and precipitation. PC1 explained over 50% of the variance in environmental variables, and the first four axes explained over 95% of the variance.

### Phenotypic measurements

We measured vegetative phenology and growth in 2010 and 2011. To measure spring bud flush, we surveyed six buds/tree every 5–10 days using a 5‐point scale (Fig. S1). This system allowed us to disentangle different aspects of the flushing process, from the initial bud swelling to final leaf emergence. For each tree, we regressed bud stage on date and interpolated the date for each tree at each stage (Fig. S2). We defined flush duration as the date of completion (stage 4) minus the date of initiation (stage 1), which represents how quickly each stem progressed through the flush process.

We surveyed fall bud set in 2010 and 2011, as the single date when the apical meristem developed bud scales that formed an acute angle (Frewen et al. 2000; Howe et al. 2000; Hall et al. 2007; Holliday et al. 2010). Surveys were conducted every 5–8 days during the fall until bud set was completed. On 17 September 2010, there was an early frost event at the UT garden. We took advantage of this event by measuring the damage to each tree. On 23 September 2010, we surveyed all trees for frost damage as the percent of meristems that had died, easily recognizable as blackened, wilting shoot tips (Fig. S3).

We measured basal diameter and height of the tallest shoot on every tree in 2010 and 2011, after growth had ceased in the fall. Baseline measurements were taken in the spring of 2010 before growth initiation. During fall 2011, the fence at our UT site was vandalized, cattle entered, and >95% of trees were damaged. Therefore, 2011 growth and bud set data were available only for the AZ and AB sites.

Our arthropod community survey was performed on 20–22 June 2011 at our UT site, the timing chosen because previous studies have demonstrated this as the peak of arthropod abundance (Wimp et al. 2005). We completely surveyed all trees visually and identified all arthropods to species when known and otherwise recognizable taxonomic units (RTUs) (Wimp et al. 2005; Bangert et al. 2006; Keith et al. 2010). We used the R package vegan (Oksanen et al. 2015) to calculate arthropod richness (S; number of different RTUs), abundance (A; total number of individual arthropods), and Shannon diversity (H'; metric that factors in the abundance of each RTU). We constructed species accumulation curves. At this same time, we measured midseason tree height, number of leaves, and SPAD (as the average from 10 leaves/tree; Spectrum Technologies, Aurora, IL), which assesses leaf chlorophyll, which is correlated with foliar nitrogen (Martin et al. 2007).

### Statistical analyses

#### Population differentiation

We compared the genetic variance within and among populations in our tree phenotypic measurements to the genetic variance at neutral genetic loci (Evans et al. 2015). For quantitative traits, a ratio of these variances may be defined as

(1) $$Q_{\mathrm{ST}}=\frac{\sigma_{P}^{2}}{\sigma_{P}^{2}+2\sigma_{G}^{2}}$$

where $\sigma_{P}^{2}$ and $\sigma_{G}^{2}$ are the between‐population and within‐population genetic variances, respectively (Spitze 1993; McKay and Latta 2002).

For each trait in each garden and each year, we estimated variance components using hierarchical Bayesian analysis (Clark and LaDeau 2004; Clark 2007) on data collected from the three local populations with genotypic replication. We first removed the within‐garden microsite heterogeneity (Zamudio et al. 2007) by fitting a thin‐plate spline surface with the *Tps* function in the *fields* package (Nychka et al. 2014) in the R statistical package. The residuals from this fitted model represent the traits after correction for spatial variation within each garden, and we used these values for subsequent analyses. The phenotype (*y*_*igp*_) of an individual tree *i* of genotype *g* from population *p* was modeled as:

(2) $$y_{igp}=\alpha_{0}+\beta_{g(p)}+\gamma_{p}+\varepsilon_{igp}$$

where *α*_0_ is the intercept (Gaussian prior *N*(0,100)), and the error *ε*_*igp*_ is Gaussian *N*(*ε*|0, $\sigma_{W}^{2}$). The random genotype (*β*_*g(p)*_) and population effects (*ϒ*_p_) are normally distributed with means of 0 and variances $\sigma_{G}^{2}$ and $\sigma_{P}^{2}$, respectively. Weak inverse gamma hyperpriors were given for the variances ($\sigma_{P}^{2}$ , $\sigma_{G}^{2}$ , and $\sigma_{W}^{2}$ ~ IG(1, 8)). The analysis was performed in *R* v. 2.14.1 (R Core Team 2014) with a Markov chain Monte Carlo method (Clark and LaDeau 2004; Clark 2007); see Appendix S1 for details of the model. Posterior distributions of all parameters were sampled using 20,000 steps through the sampler after a burnin of 10,000 steps. Convergence was assessed using visual inspection of the chains, and multiple runs resulted in very similar estimates.

Estimates of *F*_ST_ and 95% confidence intervals (CIs) were taken from a previous study of these collections consisting of 24 simple sequence repeat (SSR) loci as described previously (Evans et al. 2015), details of which can be found in the Appendix. *Q*_ST_ *–F*_ST_ comparisons were done by directly comparing the distributions – traits whose *Q*_ST_ 95% posterior credible interval did not overlap with the *F*_ST_ 95% confidence interval were considered significantly different (Spitze 1993; McKay and Latta 2002).

Simulation work has shown Bayesian estimation of *Q*_ST_ to be more accurate than other approaches (O'Hara and Merilä 2005). Our variance priors were weak, but reflect known genetic variation among individuals and populations in *Populus* (Pauley and Perry 1954; Dunlap and Stettler 1996; Hall et al. 2007; Rood et al. 2007; Grady et al. 2011; Rohde et al. 2011; Evans et al. 2014; McKown et al. 2014). Furthermore, analyses of bud set using restricted maximum likelihood (REML) indicated broad sense heritability (*H* ^*2*^) and *Q*_ST_ approached zero and one, respectively, because most of the variation exists among populations (see Results). Therefore, we stress that by specifying nonzero genotypic variance and lowering *Q*_ST_, our use of weak priors makes our test of divergent selection *conservative* as it becomes more difficult to support *Q*_ST_ > *F*_ST_. We also note that our garden design estimates *H* ^*2*^ based on additive and nonadditive genetic effects, rather than the additive genetic variance alone (*h* ^*2*^). However, simulations have shown that this approach lowers *Q*_ST_ estimates and is therefore a conservative test of *Q*_ST_ *> F*_ST_ (Goudet and Büchi 2006).

To take advantage of the entire collection of nine populations, we performed a second similar analysis using all tree genotypes with only one replicate in each garden. In this analysis, we first estimated the posterior mean *H* ^*2*^ ($=\frac{\sigma_{G}^{2}}{\sigma_{G}^{2}+\sigma_{W}^{2}}$) from the three‐population analysis described above. Variances were estimated using a similar approach, except that our model did not include the *β*_g(p)_'s, and therefore, $\sigma_{w}^{2}$ includes both the genotype‐within‐population variance and the error variance. We then estimated *Q*_ST_ among all nine populations at each site with a similar model as above, but replaced $2\sigma_{G}^{2}$ with $2H^{2}\sigma_{W}^{2}$. This assumes that the pooled within‐population genetic variance from the three local populations (using replicated tree genotypes spanning the north–south range) would be the same as the pooled nine‐population within‐population genetic variance if we were able to estimate it. This approach has been used to estimate *Q*_ST_ when no estimate of the within‐population genetic variance is available (Leinonen et al. 2008). However, our analysis differs from such studies, because it was performed using common gardens and is not confounded by environmental variation among population source locations (Lynch and Walsh 1998). We tested for population × environment interactions with a similar nine‐population model as just described, but included the garden and population × garden interaction terms (“G × E Model”).

Arthropod community diversity indices of S and A approximated a Poisson distribution, while H' was approximately normal. We analyzed H' as described above, but we estimated the genetic variance within and among populations of S and A using a generalized linear model that accounted for extra‐Poisson variability in the community phenotypes (Evans et al. 2012). Measurements of A and S were modeled as

(3) $$y_{igp}∼\text{Poisson}\left(\theta_{igp}\right)$$

where *θ*_*igp*_ is the Poisson mean for tree *i* of genotype *g* from population *p*. The tree means were modeled as:

(4) $$\text{ln}\left(\theta_{igp}\right)=\alpha_{0}+\beta_{g(p)}+\gamma_{p}+\varepsilon_{igp}$$

with parameters and their variances as described above. Again, the analysis used an MCMC method using Gibbs sampling, but with an imbedded Metropolis step evaluating the probability of the modeled tree Poisson means (Clark and LaDeau 2004; Clark 2007; Evans et al. 2012).

#### Climate analyses

We tested for environmental clines in phenological and growth traits across our collection using correlation, a common procedure implemented in other studies of forest trees (Hall et al. 2007; Holliday et al. 2010; Keller et al. 2010). Genetic correlations among traits were assessed using the genotype and population posterior mean effect estimates.

To test the hypothesis that traits with stronger climate correlations were more differentiated, we tested the correlation between the climate–trait correlation coefficient (|*r*|, from above) and *Q*_ST_ estimates across traits. We tested this using the correlation with each of the first two climate principal component axes for each trait at the population level.

To test the hypothesis that altered climate influences tree growth, we used a transfer function approach (Rehfeldt et al. 1999; O'Neill et al. 2008; Wang et al. 2010; Grady et al. 2011) to test the effect of the climate PC1 transfer distance (garden – source; pc_trd). We use the climate PC1 because it is strongly correlated with many adaptive traits and provides an overall climate (and latitude/photoperiod) measure. This approach tests for local adaptation by fitting a quadratic model (pc_trd + pc_trd^2^), and a negative squared term and apex ~0 indicates evidence of local adaptation. We applied the GxE model described above, with population, garden, and population x garden interaction terms, but included fixed effects of pc_trd and pc_trd^2^ with Gaussian priors *N*(0,100). We analyzed 2010 height growth, as tree height is commonly used as a surrogate for tests of adaptation in such contexts (Rehfeldt et al. 1999; O'Neill et al. 2008; Wang et al. 2010; Grady et al. 2011).

## Results

### Phenology and growth trait differentiation

**Figure 2:**
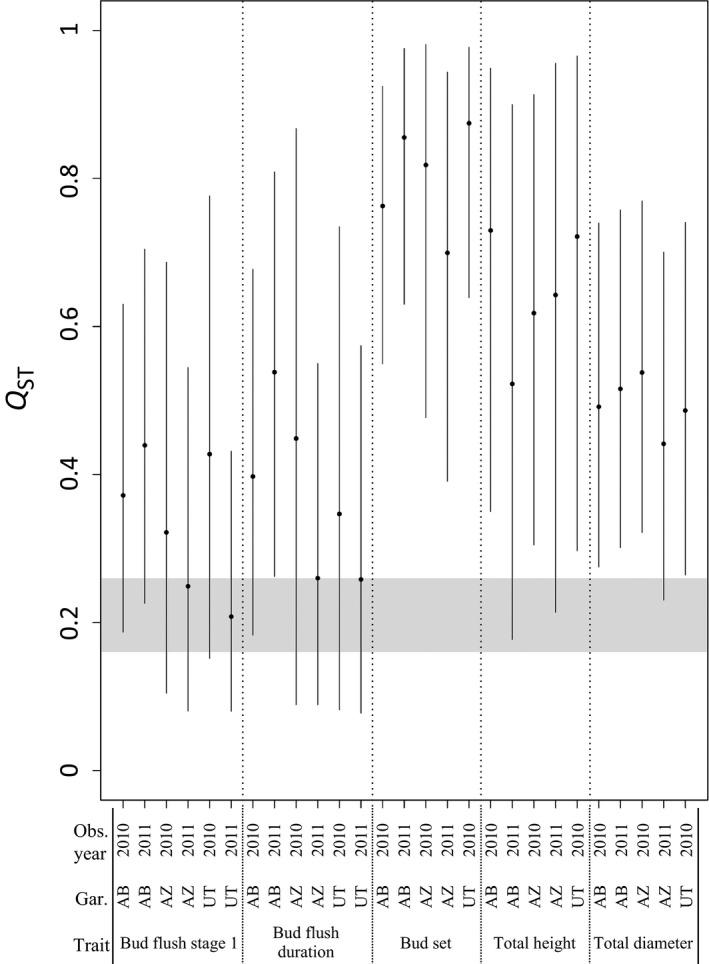
Posterior mean *Q*_ST_ and 95% CIs (points and vertical bars, respectively) for tree phenotypic traits measured in each of the three common gardens (AB, UT, and AZ), estimated from the nine‐population model. Gray shaded area represents the *F*_ST_ 95% CI.

*Q*_ST_ estimates based on the three local populations (estimating *H* ^*2*^) and those based on all nine populations (assuming an equivalent *H* ^*2*^) were generally similar although the nine‐population estimates were slightly lower. There was a greater uncertainty in the three local population *Q*_ST_ estimates, reflecting the smaller number of populations (Table 1). Because the three‐ and nine‐population estimates and inferences were similar, in the following descriptions we restrict our discussion to the *Q*_ST_ estimates from all nine populations. In comparison with *Q*_ST,_ we found that *P. angustifolia* is strongly genetically differentiated at neutral SSR loci throughout its range (*F*_ST_ (95% CI) = 0.21 (0.16–0.26; Fig. 2). These results are reported in the study of Evans et al. (2015).

**Table 1:** Quantitative trait differentiation of tree phenotypic traits in three common garden sites. Estimates and 95% credible intervals of *Q*_ST_ from both the three local populations with genotypic replication, and using the mean *H* ^*2*^ to estimate the genetic variance for all nine populations are presented. Broad sense heritability (*H* ^*2*^) pooled across the entire collection and broad sense heritability (*H* ^*2*^) pooled from the hierarchical analysis within populations are both presented

Trait	Garden	Obs. Year	*H* ^*2*^	*H* ^*2*^	*Q*_ST_	*Q*_ST_
			Collection‐wide	Hierarchical Model	3 Populations	9 Populations
Bud Flush Initiation	AB	2010	0.53 (0.42–0.65)	0.37 (0.25–0.5)	0.33 (0.1–0.71)	0.37 (0.19–0.63)
	AB	2011	0.11 (0.06–0.17)	0.11 (0.06–0.17)	0.64 (0.34–0.91)	0.44 (0.23–0.7)
	UT	2010	0.38 (0.19–0.57)	0.16 (0.05–0.34)	0.6 (0.21–0.92)	0.43 (0.15–0.78)
	UT	2011	0.41 (0.25–0.56)	0.38 (0.22–0.54)	0.23 (0.06–0.58)	0.21 (0.08–0.43)
	AZ	2010	0.37 (0.17–0.57)	0.28 (0.07–0.5)	0.22 (0.03–0.68)	0.32 (0.1–0.69)
	AZ	2011	0.3 (0.14–0.46)	0.22 (0.09–0.38)	0.38 (0.1–0.79)	0.25 (0.08–0.54)
Bud Flush Duration	AB	2010	0.37 (0.26–0.5)	0.21 (0.11–0.34)	0.4 (0.13–0.79)	0.4 (0.18–0.68)
	AB	2011	0.06 (0.03–0.1)	0.06 (0.03–0.1)	0.57 (0.26–0.88)	0.54 (0.26–0.81)
	UT	2010	0.13 (0.02–0.31)	0.1 (0.02–0.24)	0.48 (0.11–0.89)	0.35 (0.08–0.73)
	UT	2011	0.26 (0.11–0.43)	0.16 (0.05–0.31)	0.42 (0.12–0.83)	0.26 (0.08–0.57)
	AZ	2010	0.11 (0.01–0.34)	0.07 (0.01–0.25)	0.38 (0.05–0.87)	0.45 (0.09–0.87)
	AZ	2011	0.19 (0.08–0.34)	0.17 (0.07–0.32)	0.41 (0.13–0.81)	0.26 (0.09–0.55)
Bud Set	AB	2010	0.73 (0.64–0.8)	0.18 (0.08–0.3)	0.79 (0.52–0.96)	0.76 (0.55–0.92)
	AB	2011	0.6 (0.5–0.7)	0.06 (0.01–0.14)	0.89 (0.66–0.99)	0.86 (0.63–0.98)
	UT	2010	0.41 (0.26–0.56)	0.04 (0.01–0.11)	0.86 (0.58–0.98)	0.87 (0.64–0.98)
	AZ	2010	0.4 (0.28–0.53)	0.04 (0–0.12)	0.85 (0.5–0.99)	0.82 (0.48–0.98)
	AZ	2011	0.46 (0.32–0.6)	0.1 (0.02–0.23)	0.72 (0.35–0.97)	0.7 (0.39–0.94)
Height	AB	2010	0.06 (0.01–0.21)	0.03 (0.01–0.08)	0.79 (0.41–0.97)	0.73 (0.35–0.95)
	AB	2011	0.28 (0.14–0.42)	0.1 (0.01–0.24)	0.48 (0.1–0.91)	0.52 (0.18–0.9)
	UT	2010	0.27 (0.14–0.42)	0.04 (0–0.13)	0.79 (0.35–0.98)	0.72 (0.3–0.97)
	AZ	2010	0.43 (0.3–0.55)	0.09 (0.02–0.19)	0.72 (0.36–0.96)	0.62 (0.3–0.91)
	AZ	2011	0.32 (0.15–0.49)	0.06 (0–0.19)	0.72 (0.25–0.98)	0.64 (0.21–0.96)
Diameter	AB	2010	0.34 (0.21–0.48)	0.32 (0.2–0.46)	0.6 (0.3–0.89)	0.49 (0.28–0.74)
	AB	2011	0.31 (0.2–0.43)	0.27 (0.17–0.39)	0.57 (0.28–0.88)	0.52 (0.3–0.76)
	UT	2010	0.28 (0.17–0.41)	0.21 (0.13–0.33)	0.61 (0.32–0.9)	0.49 (0.26–0.74)
	AZ	2010	0.32 (0.22–0.44)	0.26 (0.17–0.36)	0.63 (0.34–0.9)	0.54 (0.32–0.77)
	AZ	2011	0.23 (0.14–0.35)	0.2 (0.12–0.31)	0.59 (0.29–0.89)	0.44 (0.23–0.7)

Much of the genetic variation in phenotypic traits was distributed among populations (Table 1, Fig. 2). For nearly all traits in all gardens across both years, point estimates of *Q*_ST_ were higher than *F*_ST_ estimates, although *Q*_ST_ and *F*_ST_ 95% credible and confidence intervals (CIs) often overlapped. Overall, average *Q*_ST_ across all traits, gardens, and both years (0.51) was well outside the *F*_ST_ CI, indicating support for the hypothesis that these growth and phenology are under divergent selection (Table 1, Fig. 2). Bud flush initiation (stage 1) and duration 95% CIs broadly overlapped the *F*_ST_ 95% CI (Table 1, Fig. 2). Although the point estimates were greater than *F*_ST_, this did not support the divergent selection hypothesis for bud flush due to the large variance in the estimates. However, estimates of river, garden, and river x garden interactions and their variances suggest that genetic and environmental influences both impact bud flush traits (Table 2, Fig. S4, Table S3).

**Table 2:** River, garden, and river × garden interaction variance component estimates (posterior mean ± 95% credible interval) from the G × E model

Trait	Year	Variance component	Proportion of total variance explained
Bud Flush Initiation	2010	River	0.161 (0.062–0.362)
		Garden	0.056 (0.021–0.133)
		River × Garden	0.14 (0.05–0.34)
	2011	River	0.134 (0.056–0.287)
		Garden	0.118 (0.048–0.268)
		River × Garden	0.1 (0.038–0.236)
Bud Flush Duration	2010	River	0.079 (0.027–0.185)
		Garden	0.063 (0.024–0.148)
		River × Garden	0.063 (0.021–0.16)
	2011	River	0.092 (0.036–0.206)
		Garden	0.067 (0.027–0.155)
		River × Garden	0.068 (0.026–0.166)
Bud Set	2010	River	0.144 (0.055–0.324)
		Garden	0.028 (0.007–0.075)
		River × Garden	0.165 (0.058–0.409)
	2011	River	0.121 (0.044–0.273)
		Garden	0.006 (0.002–0.017)
		River × Garden	0.156 (0.052–0.395)
Height	2010	River	0.086 (0.03–0.2)
		Garden	0.017 (0.004–0.045)
		River × Garden	0.086 (0.029–0.216)
	2011	River	0.058 (0.017–0.143)
		Garden	0.006 (0.002–0.016)
		River × Garden	0.067 (0.019–0.176)
Diameter	2010	River	0.309 (0.159–0.578)
		Garden	0.246 (0.127–0.472)
		River × Garden	0.284 (0.142–0.553)
	2011	River	0.169 (0.086–0.323)
		Garden	0.171 (0.085–0.338)
		River × Garden	0.191 (0.092–0.384)

Conversely, bud set was the most strongly differentiated trait with posterior mean *Q*_ST_ estimates >0.6 at all three sites in both years. There was no overlap in 95% CIs between *Q*_ST_ and *F*_ST_ (*Q*_*ST*_ ≫ *F*_ST_), which supports the divergent selection hypothesis. Again, the among‐river and river × garden variance were comparable and larger than the among‐garden variance (Table 2, Fig. S4), suggesting that genetic differences are more important than environment for bud set.

Together, our results supported the hypothesis that height and diameter are under divergent selection. Height and diameter averaged (range) 42 cm (4–110) and 0.5 cm (0.1–1.5), respectively, in Arizona in 2010; 52 cm (4–183) and 0.5 cm (0.1–3) in Arizona in 2011; 50 cm (8–130) and 0.6 cm (0.2–2) in Utah in 2010; 40 cm (3–146) and 0.5 cm (0.1–1.8) in Alberta in 2010; and 48 cm (3–184) and 0.8 cm (0.2–2.9) in Alberta in 2011 (Fig. S4). *Q*_ST_ estimates for height and diameter were typically higher than *F*_ST_ (Table 1, Fig. 2). The CIs of these growth traits were mostly nonoverlapping the *F*_ST_ CI in most locations across the 2 years. The proportions of variance explained by river, garden, and river × garden interactions were comparable for height and for diameter, suggesting that genetic, environmental, and G × E interactions are important for tree growth (Table 2, Fig. S4).

**Figure 3:**
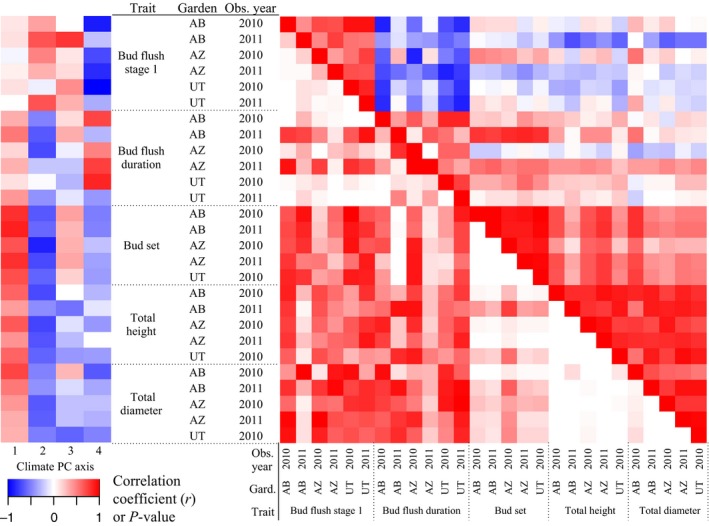
On left, correlation coefficients of each trait with the first 4 climate principal component axes. On right, pairwise trait correlations using posterior population means (above diagonal) and the *P*‐value (below diagonal) for all traits measured in each year and in each garden. See Tables S2 and S4 for values.

**Figure 4:**
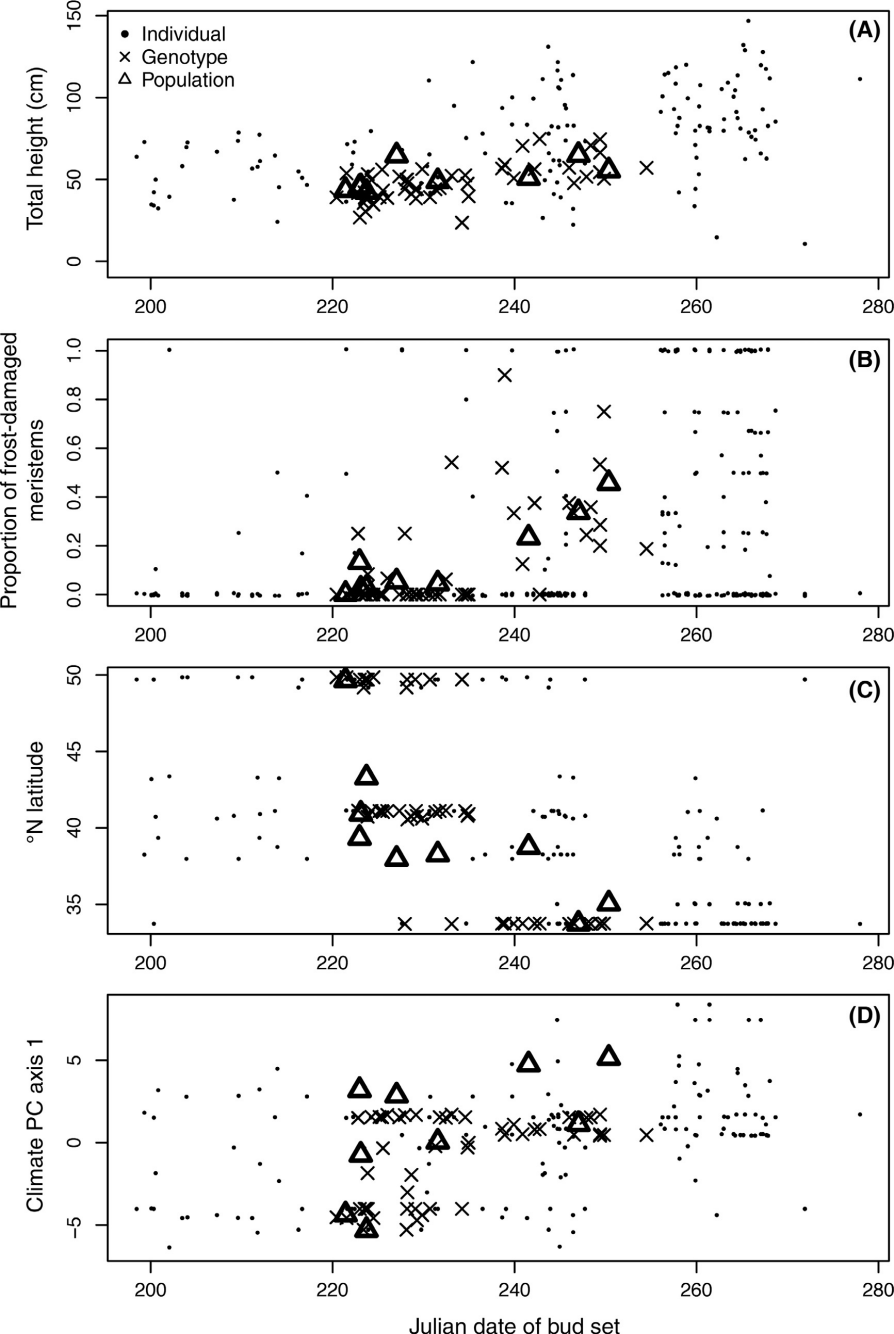
2010 bud set date (*x*‐axis) influences height (A) and frost damage (B) and is influenced by latitude (C) and climate (D).

Between bud flush initiation and its duration, correlation coefficients were high at both the population and genotypic levels at all three sites (Fig. 3, Tables S4 and S5). Trees that initiated bud flush earlier took longer to complete flushing (negative relationship). Weak or no correlations were observed between bud flush and growth metrics. Bud set was moderately correlated with bud flush and most strongly correlated with height and diameter; that is, larger trees had later bud set (Fig. 4A). Height and diameter were moderately correlated with one another. Correlations were generally stronger when traits were measured within the same garden and in the same year.

The date of bud set was correlated with frost damage in 2010 at the UT garden site. There was a strong positive phenotypic correlation between bud set date and the percent of meristems damaged (Fig. 4B, phenotypic correlation: ρ = 0.43; genotypic correlation: ρ = 0.62; population correlation: ρ = 0.93; all *P* < 0.001). Trees that set bud later were more sensitive to damage, and these were more likely to be trees transferred from southern latitude sites (Fig. 4C; bud set–latitude correlation, ρ = −0.41, *P* < 0.001). Furthermore, we observed extensive dieback of southern genotypes planted in Alberta (Fig. S3; S. B. Rood, D. W. Pearce, S. Kaluthota, pers. obs.)

### Climate analyses

Bud flush traits were moderately to strongly correlated (|*r*|>0.5) with several climate variables. These included the composite PC axes (Fig. 3) as well as degree‐day variables, the duration of the frost‐free period, and elevation (Table S5). Date of bud set varied over 60 days among individual trees, over 30 days among populations, and was also correlated with climate variables and latitude (Figs. 3, 4C,D, Table S5). In particular, it was strongly correlated with PC1, which was primarily driven by temperature variables (Table S2), to support our hypothesis that climate influences adaptive traits.

Height and diameter measurements were weakly (|*r*|<0.2) to strongly (|*r*|>0.5) correlated with climate variables, including the composite principal component scores of the first four axes. Growth showed stronger correlations with some climate variables than geographical variables, for example, degree‐days <0°C, but strong correlations with the 2nd PC axis, which was strongly determined by geographical and temperature variables.

**Figure 5:**
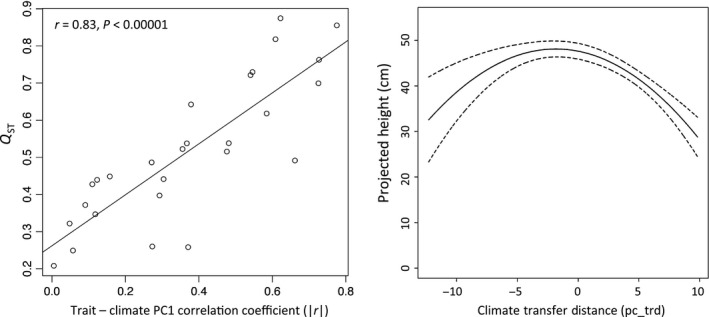
Climate influences adaptive traits. Left: Relationship between population trait differentiation (*Q*_ST_) and the strength of the correlation with climate for each trait (|*r*|). Right: Projected height (cm) across the observed range of climate PC1 transfer distances for an idealized population (random effects of river, garden, and their interactions set to 0). This indicates the expected change in growth for a fixed change in climate as measured by PC1.

Across all the traits and years, traits with the strongest climate correlations (as measured against the first two PC axes) were most differentiated (*Q*_ST_) among populations (Climate PC1: Pearson *r *=* *0.83, *P* < 0.00001; Climate PC2: Pearson *r *=* *0.56, *P* = 0.0026; Fig. 5). For example, bud set was most strongly differentiated among populations and also most strongly correlated with the climate PCs. Conversely, bud flush initiation was weakly correlated with the climate PCs and was least differentiated among populations.

Our transfer function analysis testing local adaptation identified effects of climate PC1 on 2010 height growth (posterior mean (95% CI): pc + trd = −1 (−1.57 to −0.44), pc_trd^2^ = −0.41 (−0.58 to −0.24); Fig. 5; Tables S7 and S8). In particular, the negative pc_trd^2^ term indicates that growth peaks and then declines with increasing climate transfer distance away from the source location climate, supporting our hypothesis of local adaptation. We found river, garden, and river × garden interaction effects comparable to those in the G × E model above, indicating nonclimatic environmental influences and genetic influences unrelated to tree interactions with climate (Tables S7 and S8).

### Community analyses

We observed 1016 arthropod individuals of 53 RTUs. RTUs mainly included ants (Hymenoptera: Formicidae), aphids (Hemiptera: Aphididae), and leafhoppers (Hemiptera: Cicadellidae) (Table S9). Predatory RTUs included mainly spiders (Araneae). Some RTUs were closely dependent on *P. angustifolia* (such as aphids), while others were likely incidental (e.g., mayflies (Ephemeroptera: Ephemerellidae); Table S9). The most common RTU was the aphid, *Chaitophorous populicola*. Most RTUs were rare, with only 17 observed >10 times in the garden, and the species accumulation curve did not plateau (Fig. S5). However, given that we surveyed all shoots and leaves of all trees, our sample represents an exhaustive survey of arthropods in the garden.

We found significant genetic variation in community diversity measures among and within tree populations. Community diversity measures (S, A, and H') all differed among genotypes and were heritable (posterior mean *H* ^*2*^ > 0.3; Table 3). Further, much of the genetic variation was distributed among populations. All metrics were more differentiated among populations than *F*_ST_ (Table 3), suggesting that community phenotypes are more divergent than expected.

**Table 3:** *Q*_ST_ and *H* ^*2*^ estimates of arthropod community metrics measured mid‐season in Utah, 2011. Posterior means and 95% credible intervals shown

Community phenotype	*H* ^*2*^	*Q*_ST_	*Q*_ST_
	Hierarchical model	3 Populations	9 Populations
S	0.43 (0.27–0.56)	0.65 (0.36–0.91)	0.61 (0.4–0.82)
A	0.35 (0.21–0.51)	0.63 (0.33–0.91)	0.51 (0.31–0.74)
H'	0.48 (0.25–0.64)	0.78 (0.54–0.96)	0.83 (0.69–0.93)

**Figure 6:**
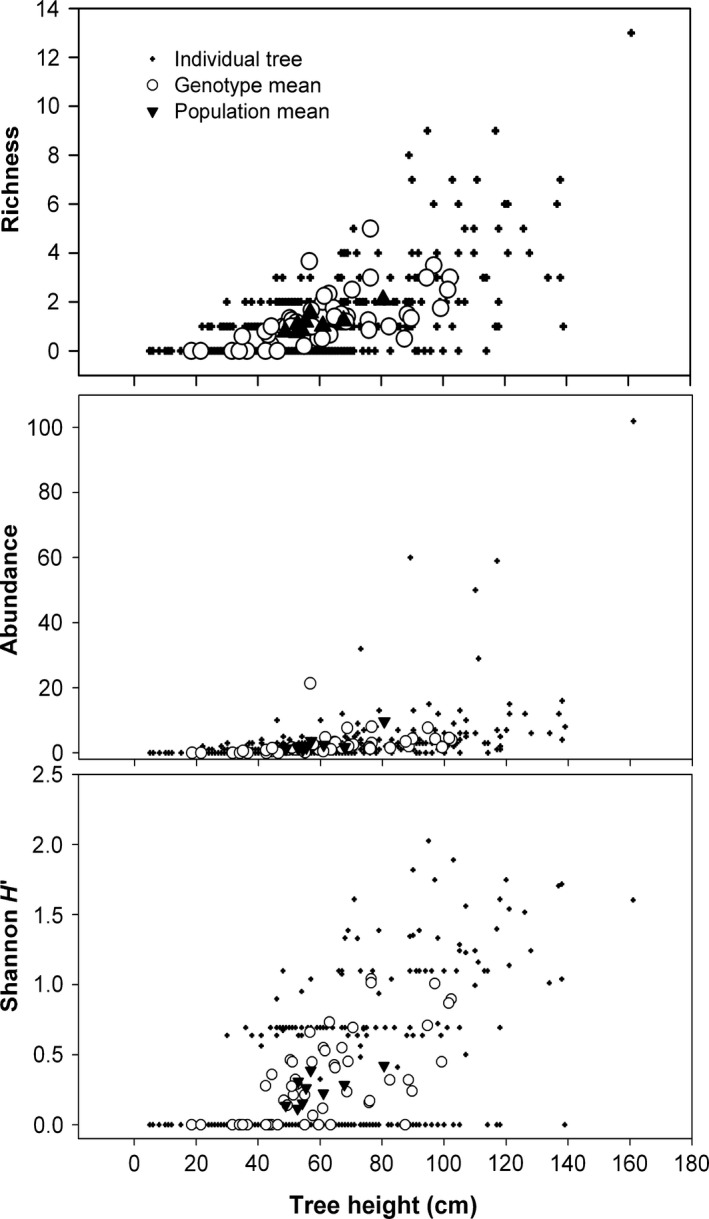
Community diversity metrics shown at the individual tree, genotype, and population levels plotted against midseason tree height in Utah in 2010.

We found that tree productivity (shoot growth) was positively correlated with community diversity metrics, supporting the hypothesis that adaptive genetic variation in *P. angustifolia* influences dependent arthropods. We found phenotypic, genotypic, and population correlations of S, A, and H' with height, number of leaves, and later flush stages and duration (Table 4, Fig. 6). No community diversity metrics were correlated with the SPAD assessments of foliar chlorophyll and nitrogen. Trees that finished bud flush later were taller than those that completed flush more quickly or finished sooner. These same tall trees supported more associated arthropods (Fig. 6).

**Table 4:** Correlations (S and A: Spearman rank; H': Pearson) between community diversity metrics and plant phenotypic traits measured mid‐season at the UT site, 2011

Plant phenotype	Individual phenotypic correlation			Genotypic correlation			Population mean correlation		
	S	A	H'	S	A	H'	S	A	H'
BF initiation	−0.07	−0.09	−0.02	−0.05	−0.27	0.07	−0.07	0.33	0.25
BF duration	0.20***	0.22***	0.22***	0.41**	0.47**	0.22	−0.15	−0.40	−0.22
Height	0.51***	0.52***	0.56***	0.71***	0.79***	0.62***	0.82**	0.72*	0.68*
No. of Leaves	0.50***	0.50***	0.61***	0.68***	0.70***	0.65***	0.58	0.60	0.67*
SPAD	0.02	0.00	0.00	0.00	−0.14	−0.01	0.47	0.33	0.41

***P < 0.001, **P < 0.01, *P < 0.05.

## Discussion

### Divergent selection in *Populus angustifolia*

Phenological traits in many forest tree species are spatially divergent and have climatic correlations (Howe et al. 2003; Savolainen et al. 2007). Using two different tests (*Q*_ST_ vs. *F*_ST_ and clinal analyses), our data support the hypothesis that bud set is genetically based and under divergent selection in *P. angustifolia*. When grown in three different common garden locations spanning the range of the species, trees from northern and colder environments set bud earlier than those from southern and warmer environments. Growth cessation and bud formation are initiated by short days, with full cold acclimation following low temperatures (Howe et al. 2003). Trees from colder source locations set bud earlier than southern trees, because they are adapted to the critical day length of northern climates, where freezing temperatures arrive earlier (i.e., longer days) in the fall. These same trees experienced much lower frost damage at our Utah site, demonstrating the adaptive nature of the tradeoff between growth and frost avoidance seen in many species of forest trees, for example, in *Picea*,* Pinus,* and *Populus*, where the timing of bud set shows latitudinal clines (Pauley and Perry 1954; García‐Gil et al. 2003; Hall et al. 2007; Holliday et al. 2010; Rohde et al. 2011; Evans et al. 2014; McKown et al. 2014; Grady et al. 2015). These and the present study suggest that climate is a key determinant of bud set and local adaptation, with a proximate cue being latitude‐driven critical photoperiod (Howe et al. 2003; McKown et al. 2014).

We split the process of bud flush into its components to estimate both its initiation and the length of time to complete the process. The lower differentiation of bud flush relative to bud set matches findings from other forest trees (Campbell 1979; Howe et al. 2003; Hall et al. 2007). However, in contrast to these studies, we found little evidence of spatially divergent selection on bud flush traits using *Q*_ST_ *–F*_ST_ comparisons. Conversely, the date at which the frost‐free period begins was more strongly correlated with bud flush than any other variable, including latitude, the major axis of neutral structure in the species (Evans et al. 2015). Bud flush is also strongly correlated with climate variables in the related species *Populus trichocarpa* Torr. & A. Gray ex Hook. (Evans et al. 2014; McKown et al. 2014) and *P. fremontii*, (Grady et al. 2015). Thus, while the among‐population component of bud flush variation is no more differentiated than the strong neutral expectations, the relationships with climate variables suggest that bud flush is under temperature‐driven cues that vary throughout the range of *P. angustifolia*, consistent with the strong influence of climate on bud flush in forest trees in general. However, as it is also more directly related to cumulative degree‐days than bud set (Howe et al. 2003), it is likely to be more plastic in response to altered conditions. Indeed, the date of bud flush in many species has been observed to shift over recent decades (Parmesan 2006).

This comparison points to the difficulty in identifying signatures of selection using comparative tests when neutral structure is strong. Many forest trees have weak neutral population structure compared with *P. angustifolia* (Howe et al. 2003; Hall et al. 2007; Holliday et al. 2010), and evidence of divergent selection may be more easily supported. For example, Hall et al. (2007) found that among a latitudinal collection of *P. tremula*,* F*_ST_ ~ 0.01. They found evidence of divergent selection in the date of bud flush and growth characters, although their estimates of *Q*_ST_ were similar to ours. Although environmental variation may influence a trait, if neutral structure covaries with that gradient due to historical and demographic factors (e.g., Evans et al. 2015), differentiating selection from other factors may be difficult. A further complication in our study is the small number of populations (*N* = 9), resulting in larger *Q*_ST_ credible intervals (O'Hara and Merilä 2005). Despite this difficulty, the connections among growth, phenological, and frost damage traits suggest that adaptive variation exists throughout the range of *P. angustifolia*, likely driven by climate.

Growth measures (end‐of‐season height and diameter) showed patterns of strong differentiation and correlations with climate variables, supporting our hypothesis of divergent selection (*Q*_ST_ *> F*_ST_). The latitudinal and temperature correlations (particularly degree‐day variables), and negative squared climate transfer distance term in our transfer function analysis suggest that these are related to climate. The genotypic correlations of flush and set with height and diameter may explain this finding, with southern, taller genotypes ceasing growth later in the fall, consistent with studies in other species (Howe et al. 2003; Hall et al. 2007; Holliday et al. 2010). The greatest growth occurred for genotypes planted slightly north of their source location (e.g., Indian Creek, UT moved ~300 km north to the UT planting site). This increase in the performance of moved populations is also consistent with the hypothesis that climate has already shifted to a more southerly climate resulting in their greater performance (e.g., Brusca et al. 2013). These relocated genotypes benefitted from longer day lengths and were more tolerant of cold temperatures than their more southern counterparts.

Decreasing growth with greater climate transfer distance is a hallmark of local adaptation (Rehfeldt et al. 1999; O'Neill et al. 2008; Wang et al. 2010). Our results (Fig. 5) indicate that as the environment shifts, due to either movement of trees or possibly over time, growth will be altered. The apex of the curve is slightly negative, which may reflect either a lag in adaptation or the relationship between movement north and longer day lengths as noted above. Because latitude and climate strongly covary in our sample, we cannot disentangle these effects. However, our results are suggestive of a strong role of climate in tree growth, as has been found in other systems and can provide guidance for restoration and forest plantation projects in choosing the most productive source locations for a given plantation location (Rehfeldt et al. 1999; O'Neill et al. 2008; Wang et al. 2010; Grady et al. 2011).

### Community ecology implications

We found that differentiation among tree populations in community phenotypes (species richness [S], abundance [A], and diversity [H']) was strong and larger than the neutral expectation (Table 3), suggesting that arthropods are responding to a tree trait(s) under divergent selection among populations. However, there is still substantial residual variation, suggesting that arthropod diversity metrics are influenced by environment as well. Arthropod diversity was positively related to tree height, number of leaves, and also duration of bud flush, which together can be viewed as a summary of tree productivity. Because tree growth patterns can be a primary driver of arthropod abundances (Price 1991, 2003; Ikeda et al. 2014), differentiation of community phenotypes among *P. angustifolia* populations may reflect differentiation of tree growth traits, and the phenological traits that influence them. These are traits that may be influenced by climate‐driven selection, as duration of flush shows some evidence of selection and is correlated with mid‐season tree height at our UT garden. Therefore, climate‐driven selection on tree phenology and growth has the potential to alter associated arthropod communities.

Productivity and phenology are not the only traits likely to impact arthropods. Genetic variation in community phenotypes can reflect variation in foliar chemistry (Bailey et al. 2006; Bangert et al. 2006; Barbour et al. 2009) in which closely related tree genotypes support a similar suite of phytochemicals, which in turn support a similar community of arthropods relative to more distantly related genotypes of the same tree species. In a study of eight geographical races of *Eucalyptus globulus* Labill., Barbour et al. (2009) found both leaf morphology and chemistry influenced a diverse community of arthropods and fungi. Thus, factors that affect these traits (such as climate) have the potential to alter dependent communities. Although we did not measure foliar chemistry, it is possible that the elevated community metric *Q*_ST_ estimates we observed are driven by other tree traits (e.g., defensive chemistry). In addition to plant traits, arthropods are impacted directly by climate, and the direct impacts of climate change on arthropods are well established (Parmesan 2006) with direct environmental impacts can be altered by indirect interactions with other strongly associated species (Evans et al. 2011). Complex interactions among climate, foundation species, and dependent organisms are likely to alter diversity patterns; however, productivity and diversity are positively correlated and this relationship has the potential to impact ecological communities under changed climate (Ikeda et al. 2014).

### Management implications

Climate is a major determinant of species' distributions and structures variation within species as an agent of natural selection. Understanding how and which traits are impacted by climate has applications such as choosing appropriate sources for tree breeding and reforestation programs (Johnson and Sorensen 2004; Difazio et al. 2011) and can inform the conservation management of forest trees in the face of ongoing climate change (Aitken et al. 2008; O'Neill et al. 2008; Wang et al. 2010; Grady et al. 2011, 2013). Phenology is clearly altered by climate change (Parmesan 2006), but one important conclusion from our study is that the impacts may be different for different phenological traits. For example, although spring flush is genetically based and shows some evidence of climate‐driven selection, local adaptation may not strongly impede this species' ability to cope with climate change, because (1) there appears to be considerable environmental influence, and (2) the proximate cues are climate variables. This is consistent with the widely observed advancement in the timing of spring bud flush (Parmesan 2006).

Alternatively, although warmer temperatures are persisting later in the fall (Parmesan 2006), the strong differentiation among populations in bud set may impede a species' ability to cope with these changes. In particular, because growth cessation is initiated by photoperiod (Pauley and Perry 1954; Howe et al. 2003; McKown et al. 2014), which is not changing, populations may not be able to extend their growth farther into fall. Over time, bud set may experience climate change‐driven response to selection, but examples of photoperiodic‐cued traits evolving in response to climate change in plants have not been reported to our knowledge. Selection driven by changes in photoperiodic cues, however, has been identified in short‐lived arthropods (Bradshaw et al. 2006).

Because climate strongly influences productivity, seed transfer zones have commonly been used to match plantation location to source climate tolerance (Johnson and Sorensen 2004; Bradley St Clair and Howe 2007; O'Neill et al. 2008). However, under forecasted climate change, relocating genotypes to increase genetic diversity in populations may provide a means to buffer against future climate change (O'Neill et al. 2008; Wang et al. 2010; Grady et al. 2011; Hoffmann and Sgrò 2011). Given the potential phenological mismatch of plants that are translocated over large latitudinal distances, management of natural and breeding populations should account for both geography and climate when considering seed sources. Movement of plants from similar or slightly southern latitudes north, while combining sources from current as well as predicted climates, will help ensure a diversity of genotypes containing variation, which may be adaptive to a range of climate outcomes (Grady et al. 2011). For example, Grady et al. (2015) has suggested a stepwise assisted migration, to first introduce genotypes adapted to short‐term climate change, while planning for additional, future introductions of genotypes adapted to predicted longer‐term changes. The reasoning behind a phased approach is that planting for projected long‐term conditions would represent a “bridge too far” and the more southerly populations could not survive the current conditions of northern sites. Because tree productivity is a determinant of arthropod diversity, factors influencing tree growth will impact other dependent species. In fact, such a relationship suggests that if climate impacts trees directly, then there will be cascading influences on communities that should be considered when managing populations in the face of climate change (Ikeda et al. 2014).

Such a strategy may be particularly important in species with strong neutral genetic structure, because, as in *P. angustifolia,* large geographical barriers among genetic groups or low gene flow will limit the natural spread of adaptive alleles across populations. However, the targeted movement of individuals from sources to match climate predictions across these barriers could buffer productivity changes predicted under altered climate for many species (Rehfeldt and Crookston 2006; Aitken et al. 2008; O'Neill et al. 2008; Wang et al. 2010; Grady et al. 2011; McLane et al. 2011; Leites et al. 2012). Our study suggests that species like *P*. *angustifolia*, which show divergent selection for key phenological traits across its range and strong genetic structure, may benefit from targeted movement of adaptive genotypes.

## Data accessibility

All data has been deposited in the Dryad digital repository. URL: http://dx.doi.org/10.5061/dryad.ch720.

## Conflict of Interest

None declared.

## Supporting information


**Appendix S1.** Hierarchical Bayesian model description and details
**Figure S1.** Vegetative bud flush scale used to measure spring phenology.
**Figure S2.** Plots of observed bud flush stage and Julian date.
**Figure S3.** Frost damage to *P. angustifolia*.
**Figure S4.** Posterior predicted effects of tree populations in each garden for all traits.
**Figure S5.** Species abundance and accumulation curves for UT arthropod surveys.
**Table S1.** Sample sizes after initial planting and mortality.

**Table S2.** PCA loadings of the 24 climate and geography variables.
**Table S3.** Posterior estimates of river, garden, and river x garden interaction effects from the G × E model.
**Table S4.** Pairwise trait correlations using posterior population means.
**Table S5.** Pairwise trait correlations using posterior genotype means.
**Table S6.** Correlations of trait posterior genotype means and climate and geography variables.
**Table S7.** Posterior effect estimates from the climate transfer function.
**Table S8.** Posterior variance estimates from the climate transfer function.
**Table S9.** List of recognizable taxonomic units (RTUs).
