# Supplementary material for: Bud phenology and growth are subject to divergent selection across a latitudinal gradient in Populus angustifolia and impact adaptation across the distributional range and associated arthropods
Source: Ecol Evol. 2016 Jun 10;6(13):4565–81. doi: 10.1002/ece3.2222 (PMC4931002; doi:10.1002/ece3.2222)
Supplement: Supplementary file 1 — Appendix S1. Hierarchical Bayesian model description and details Figure S1. Vegetative bud flush scale used to measure spring phenology. Figure S2. Plots of observed bud flush stage and Julian date. Figure S3. Frost damage to P. angustifolia. Figure S4. Posterior predicted effects of tree populations in each garden for all traits. Figure S5. Species abundance and accumulation curves for UT arthropod surveys. Table S1. Sample sizes after initial planting and mortality. [file ECE3-6-4565-s001.docx]

Appendix S1

*F_ST_* calculations:

To estimate neutral population structure, we used results reported from Evans et al. (2015) for 24 SSR loci. These loci were mostly tri-and tetra-nucleotide repeats, identified originally from the *P. trichocarpa* genome (Tuskan et al. 2006), which were variable in *P. angustifolia*. Full summary and descriptive statistics for all loci can be found in Evans et al. (2015). Evans et al. (2015) used analysis of molecular variance (AMOVA) among source rivers in Arlequin v.3.1 (Excoffier and Lischer, 2010) to estimate *F_ST_*, the proportion of molecular variance at SSR loci among rivers. 95% confidence intervals were estimated using 20,000 bootstrap replicates resampled over loci. Evans et al. (2015) also assessed *F_ST_* using randomly-chosen sequence fragments, which had very similar *F_ST_* and 95% CI estimates.

The model:

We used a hierarchical Bayesian model (Clark and LaDeau 2004, Clark 2007, Evans et al. 2012) to estimate parameters and variance components for tree phenotypic measurements and Shannon H’ (arthropod community surveyed on each tree).

*Regression parameters:*

The fixed effect regression parameter (α_0_ for the intercept) was sampled from the conditional posterior distribution

p(α | Y, β, ϒ, σ^2^_G_, σ^2^_P_, σ^2^_w_, **X**, **a**, V_α_, *a_G_, b_G_*, *a_P_, b_P,_ a_w_, b_w_* )

∝ N(Y | **Xa** + β*_g(p)_* + ϒ*_p_*, σ^2^_w_) x N_4_(α | a_α_, V_α_)

= N(α |Vv,V)

where

V^-1^ = 1/(σ^2^_w_ + σ^2^_G_ + σ^2^_P_) * **X**^T^**X** + V_α_^-1^

and

v = 1/(σ^2^_w_ + σ^2^_G_ + σ^2^_P_) * **X**^T^Y + V_α_^-1^ a_α_

Updated values of α were drawn from the multivariate normal N_4_(α|Vv,V)

*Random population effects:*

Random population effects (ϒ*_p_*) were sampled from the conditional posterior distribution

p ϒ*_p_* | Y, β, ϒ, σ^2^_G_, σ^2^_P_, σ^2^_w_, **X**, **a**, V_α_, *a_G_, b_G_*, *a_P_, b_P,_ a_w_, b_w_* )

∝ N(Y | **Xa** + β*_g(p)_* + ϒ*_p_*, σ^2^_w_) x N(ϒ*_p_* | 0, σ^2^_P_)

= N(ϒ*_p_* | V*_p_*v*_p_*, V*_p_*)

where

V*_p_*^-1^ = nobsp/σ^2^_W_ + 1/σ^2^_P_

and

v*_p_* = 1/σ^2^_w_ ∑ (y*_igp_* – **X_i_a_i_** - β*_g(p)_*)

Updated values of ϒ*_p_* were drawn from normal N(ϒ*_p_* | V*_p_*v*_p_*, √V*_p_*)

*Random genotype effects:*

Random genotype effects nested within populations (β*_g(p)_*) were sampled from the conditional posterior distribution

p(β*_g(p)_* | Y, β, ϒ, σ^2^_G_, σ^2^_P_, σ^2^_w_, **X**, **a**, V_α_, *a_G_, b_G_*, *a_P_, b_P,_ a_w_, b_w_* )

∝ N(Y | **Xa** + β*_g(p)_* + ϒ*_p_*, σ^2^_w_) x N(β*_g(p)_* | 0, σ^2^_G_)

= N(β*_g(p)_* | V*_g(p)_*v*_g(p)_*, V*_g(p)_*)

where

V*_g(p)_*^-1^ = nobsg/σ^2^_W_ + 1/σ^2^_G_

and

v*_g(p)_* = 1/σ^2^_w_ ∑ (y*_igp_* – **X_i_a_i_** - ϒ*_p_*)

Updated values of β*_g(p)_* were drawn from normal N(β*_g(p)_* | V*_g(p)_*v*_g(p)_*, √V*_g(p)_*)

The residual variance σ^2^_w_ was sampled from the posterior

p(σ^2^_w_ | Y, β, ϒ, σ^2^_G_, σ^2^_P_, σ^2^_w_, **X**, **a**, V_α_, *a_G_, b_G_*, *a_P_, b_P,_ a_w_, b_w_* )

∝ N(Y | **Xa** + β*_g(p)_* + ϒ*_p_*, σ^2^_w_) x IG(σ^2^_w_ | *a_w_, b_w_*)

= IG(σ^2^_w_ | s1, s2), where

s1 = *a_w_* + n/2

and

s2 = *b_w_* + (**Y - Xa - β*_g(p)_* - ϒ*_p_***)^T^(**Y - β*_g(p)_* + ϒ*_p_***)/2

The variance of the population random effects, σ^2^_P_, was sampled from the posterior

p(σ^2^_P_ | Y, β, ϒ, σ^2^_G_, σ^2^_P_, σ^2^_w_, **X**, **a**, V_α_, *a_G_, b_G_*, *a_P_, b_P,_ a_w_, b_w_* )

∝ N(Y | **Xa** + β*_g(p)_* + ϒ*_p_*, σ^2^_w_) x IG(σ^2^_P_ | *a_P_*, *b_P_*)

= IG(σ^2^_P_ |v1, v2),

where

v1 = *a_P_* + #populations/2

and

v2 = *b_P_* + ½ ∑ ϒ*_p_* ^2^

The variance of the genotype within population random effects, σ^2^_G_, was sampled from the posterior

p(σ^2^_G_ | Y, β, ϒ, σ^2^_G_, σ^2^_P_, σ^2^_w_, **X**, **a**, V_α_, *a_G_, b_G_*, *a_P_, b_P,_ a_w_, b_w_* )

∝ N(Y | **Xa** + β*_g(p)_* + ϒ*_p_*, σ^2^_w_) x IG(σ^2^_G_ | *a_G_*, *b_G_*)

= IG(σ^2^_G_ |v1, v2),

where

v1 = *a_G_* + #genotypes/2

and

v2 = *b_G_* + ½ ∑ β*_g(p)_*^2^

Model convergence was assessed by visual inspection of Gibbs chains. Multiple chains were run using different starting conditions, and convergence to the same parameter estimates was achieved.

Figure S1. Vegetative bud flush scale used to measure spring phenology.


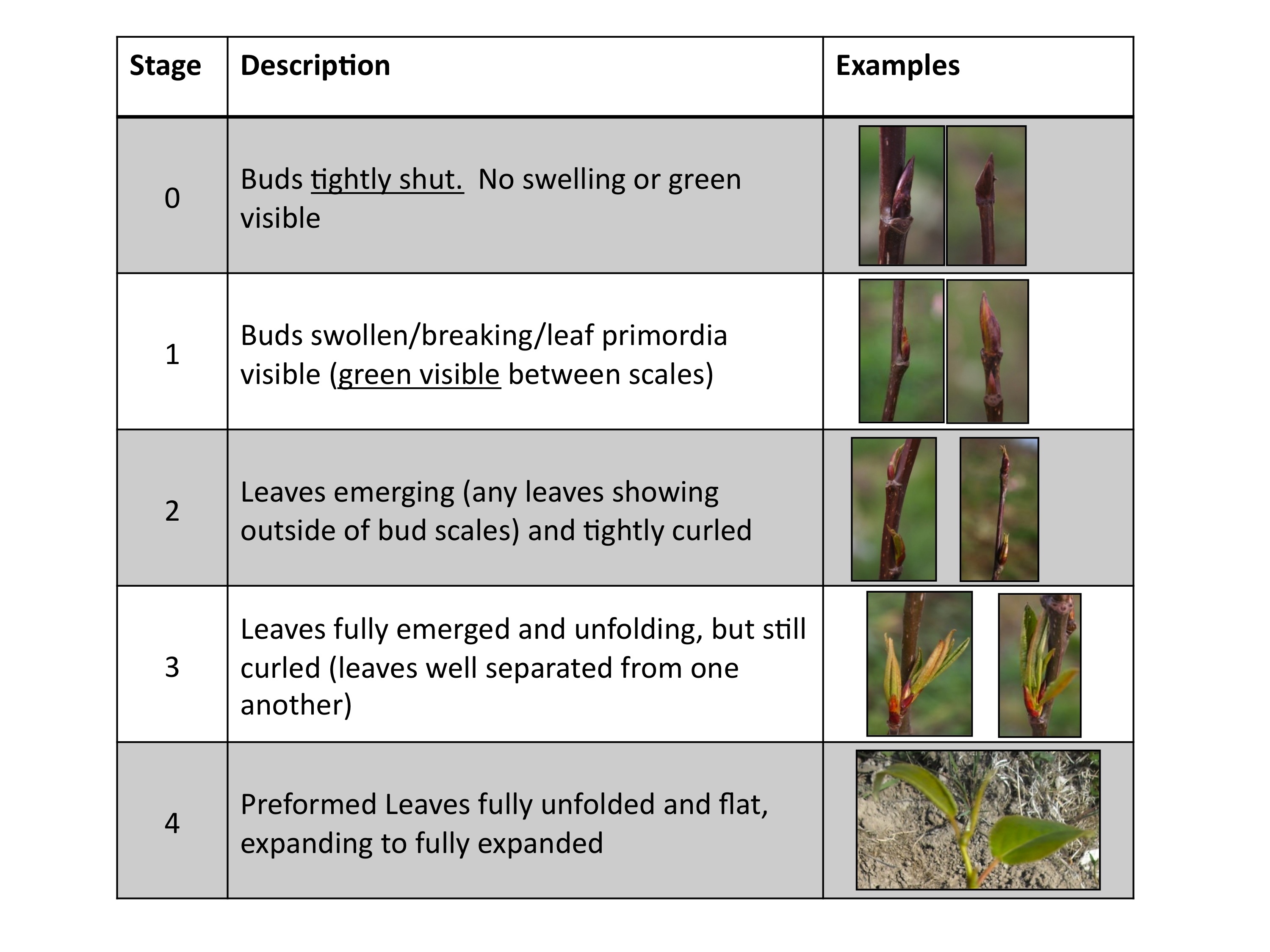
Figure S2. A random sample of stems from the AZ site in 2010, with 6 buds/tree/date surveyed, and the fitted regression line used for interpolation of flush stage and duration.

Figure S3. Example of frost damaged (top left) and undamaged (top right) shoots on 23 September 2010, after the 17 September 2010 frost event. Example of extensive dieback of an Arizona genotype planted in Alberta, resulting in bushy growth forms (bottom; photo: S. Rood).


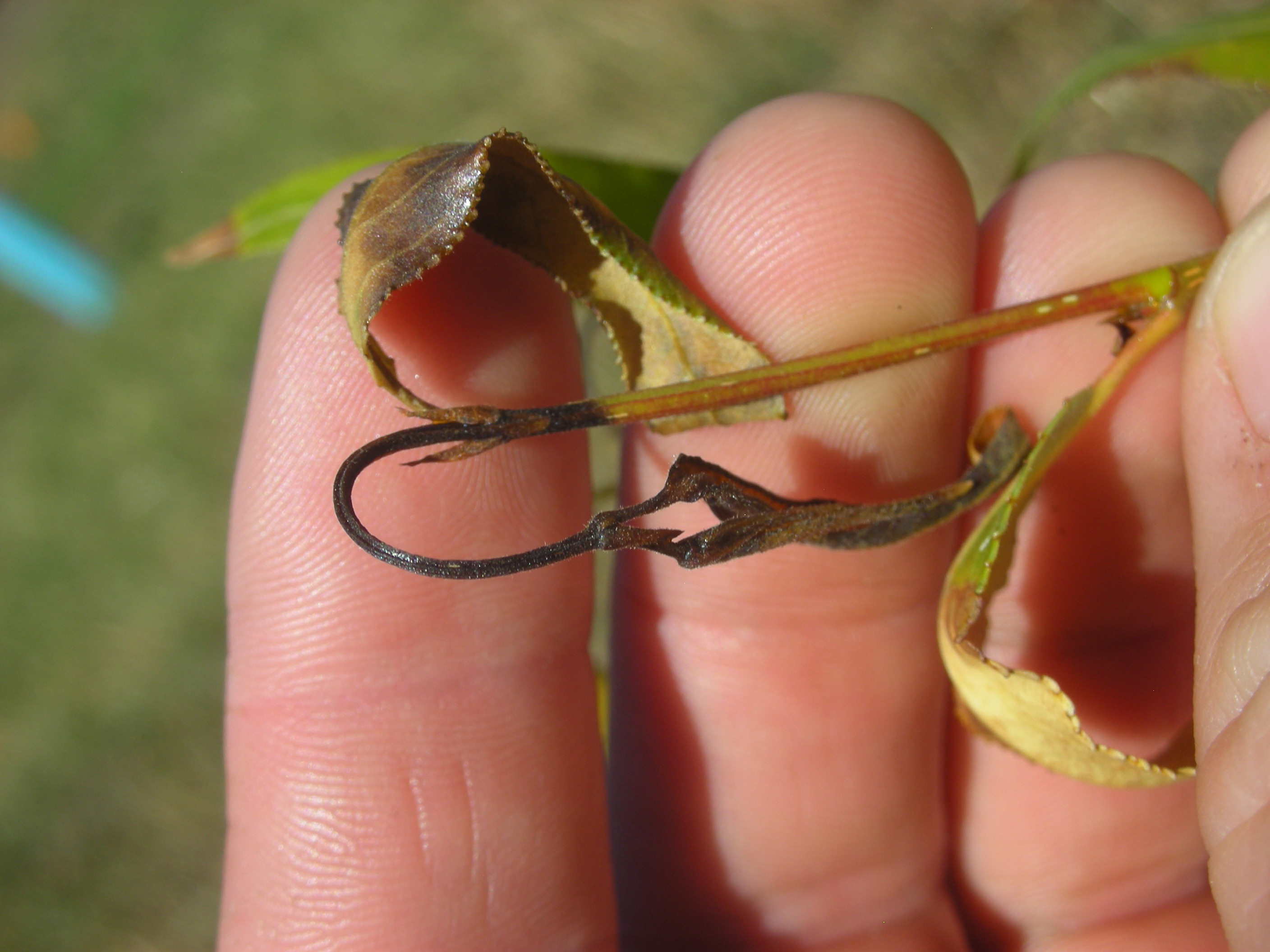


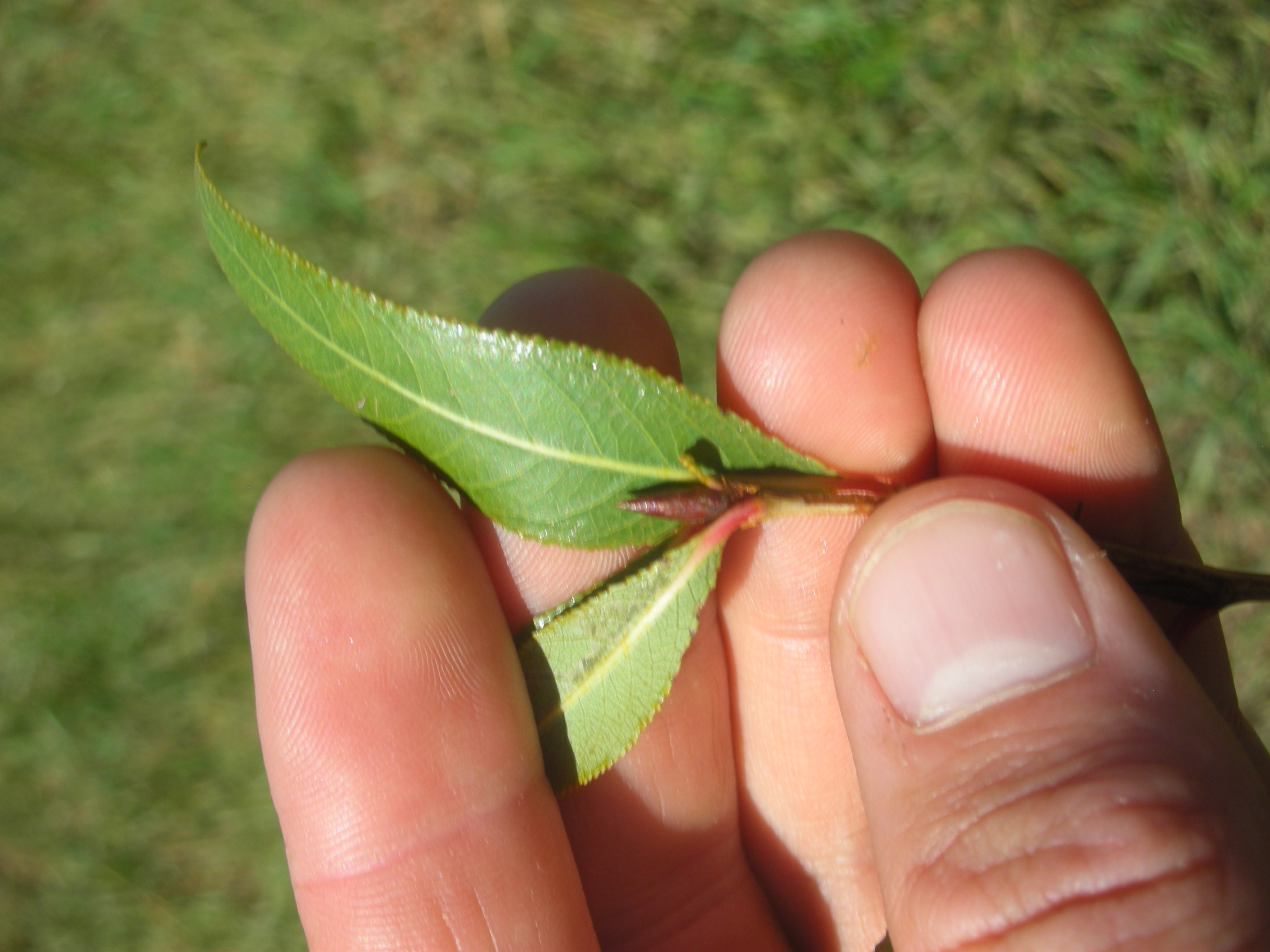


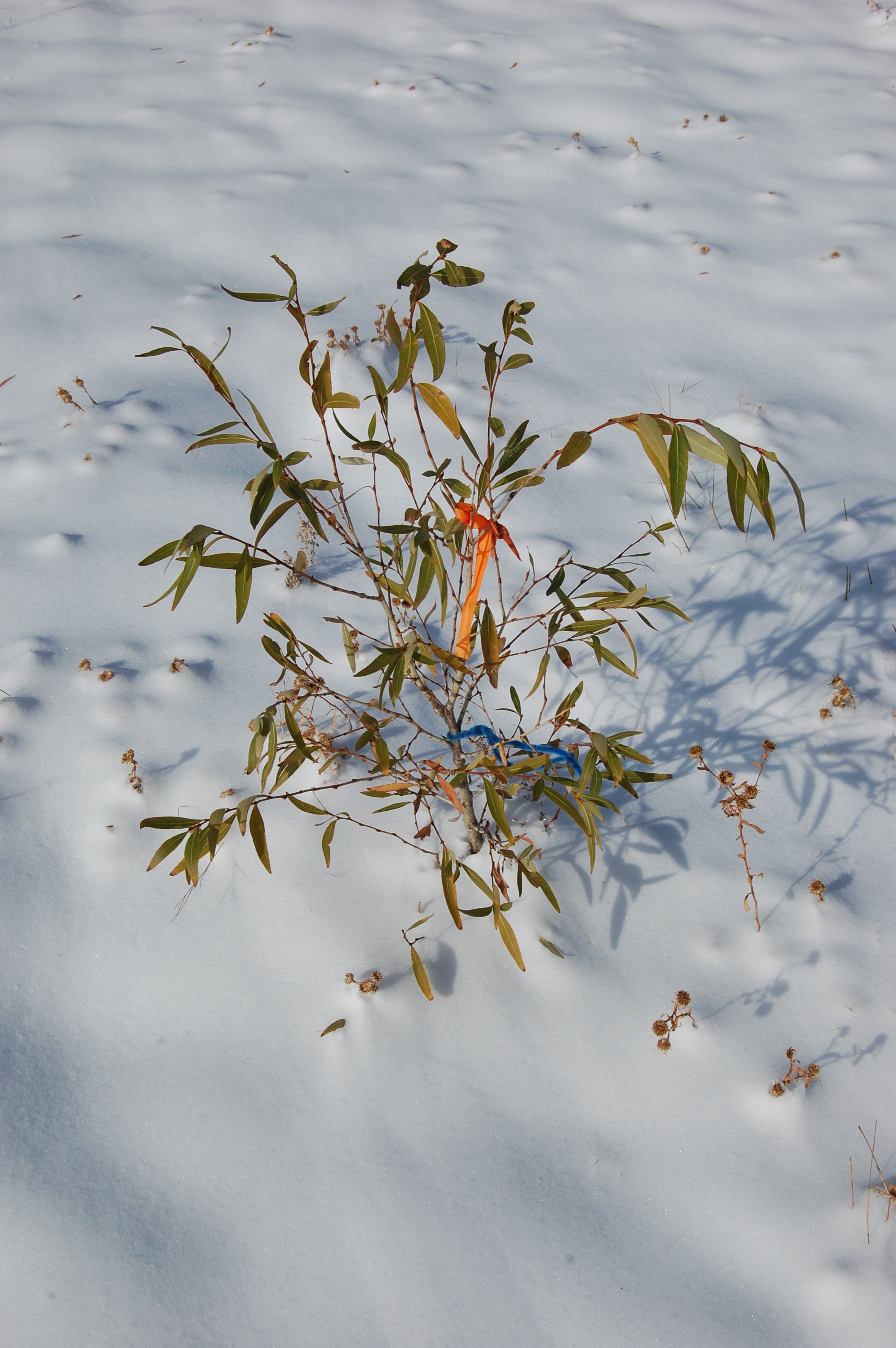


Figure S4. Significant river, garden, and river x garden interaction effects influence adaptive traits. Symbols represent different gardens, colors represent years. Posterior mean +/- 95% credible intervals are shown.


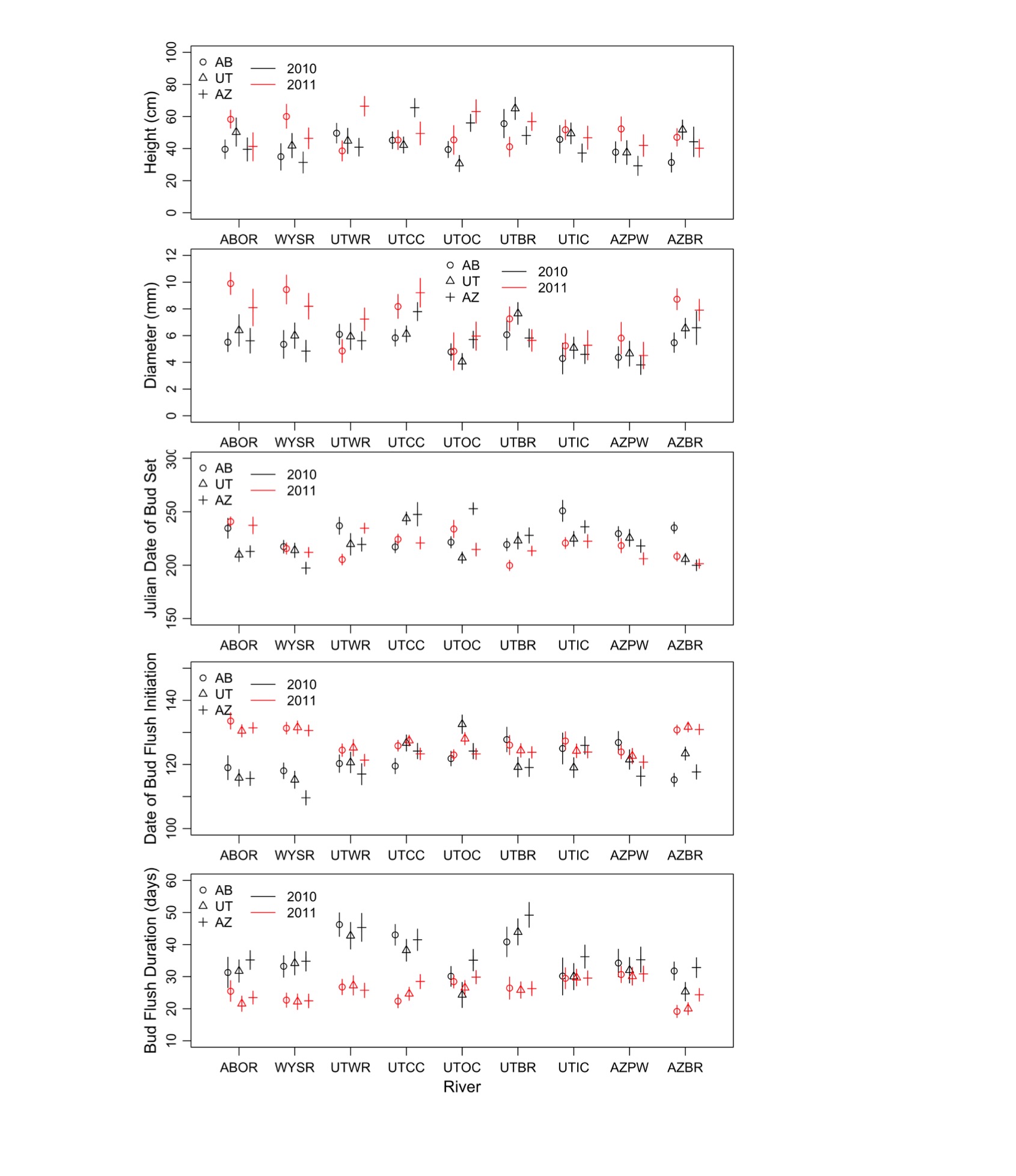


Figure S5. Species abundance curve (top) and accumulation curve (bottom) for midseason arthropod surveys at the UT site in 2011. Dashed lines in the accumulation curve represent the 95% confidence interval from 1000 bootstrap replicates sampling trees with replacement.

Table S1. Sample sizes after initial planting and mortality. Subsequent winter mortality and replanting resulted in altered sample sizes for different trait measurements.

| Reps/Geno | River | # Genotypes | | |
| --- | --- | --- | --- | --- |
|  |  | AB Site | UT Site | AZ Site |
| 2+ | Oldman, AB | 22 | 8 | 10 |
| 2+ | Blue, AZ | 18 | 19 | 15 |
| 2+ | Weber, UT | 21 | 14 | 15 |
| 1 | Oldman, AB | 34 | 26 | 15 |
| 1 | Blue, AZ | 22 | 15 | 19 |
| 1 | Pumphouse Wash, AZ | 11 | 12 | 5 |
| 1 | Beaver, UT | 46 | 24 | 26 |
| 1 | Corn Creek, UT | 32 | 26 | 15 |
| 1 | Indian Cree, UT | 29 | 19 | 20 |
| 1 | Oak Creek, UT | 26 | 17 | 19 |
| 1 | Weber, UT | 29 | 21 | 24 |
| 1 | Snake, WY | 44 | 16 | 21 |

Tables S2-S9 are provided in the online supplementary file.
